# Food Insecurity in Undergraduates During and After Remote Learning: A Brazilian Multicenter Study

**DOI:** 10.3390/ijerph22101508

**Published:** 2025-09-30

**Authors:** Liana Galvão, Luana Ataliba, Jussara Oliveira, Doroteia Höfelmann, Sandra Crispim, Alanderson Ramalho, Fernanda Martins, Bartira Gorgulho, Paulo Rodrigues, Clélia Lyra, Betzabeth Slater, Dirce Marchioni, Bruna Maciel

**Affiliations:** 1 Graduate Program in Health Sciences, Federal University of Rio Grande do Norte, Natal 59078-970, Brazil; liana.galvao.017@ufrn.edu.br; 2 Department of Nutrition, Federal University of Rio Grande do Norte, Natal 59078-970, Brazil; luana.ataliba.701@ufrn.edu.br (L.A.); jussara.oliveira.116@ufrn.edu.br (J.O.); clelia.lyra@ufrn.br (C.L.); 3 Department of Nutrition, Federal University of Paraná, Curitiba 80210-170, Brazil; doroteia.hofelmann@ufpr.br (D.H.); crispim@ufpr.br (S.C.); 4 Graduate Program in Public Health, Federal University of Acre, Rio Branco 69920-900, Brazil; alanderson.ramalho@ufac.br (A.R.); fernanda.martins@ufac.br (F.M.); 5 Department of Food and Nutrition, Federal University of Mato Grosso, Cuiabá 78060-900, Brazil; bartira.gorgulho@gmail.com (B.G.); prmr84@gmail.com (P.R.); 6 Department of Nutrition, University of São Paulo, São Paulo 01246-904, Brazil; bslater@usp.br

**Keywords:** sociodemographic factors, body weight changes, diet quality, perceived stress, college students

## Abstract

Objective: This study aimed to evaluate food insecurity and associated factors during and after remote learning among undergraduates at Brazilian public universities. Methods: This is a comparative study of two cross-sectional studies; the first had its data collection from August 2020 to February 2021, and the second from May 2023 to December 2023. The questionnaire contained socio-economic variables, the Brazilian Food Insecurity Scale, the Diet Quality Scale, and the Perceived Stress Scale. Results: In total, 4799 undergraduates of Brazilian public universities responded in the first study, and 2897 responded in the second. Food insecurity was present in 36.5% of the students in 2020/2021 and 35.9% of the students in 2023. In the correspondence analysis, low income, poor health, stress and poor diet were associated with food insecurity in 2020/2021. Low income, reduced income, poor health, stress and diet quality were associated with food insecurity in 2023. Logistic regressions demonstrated that the year of collection, whether during or after remote learning, did not significantly contribute to food insecurity. However, students from low-income families had the highest AOR for food insecurity; no change in income or weight and lower perceptions of stress were associated with a lower AOR for food insecurity.

## 1. Introduction

Food insecurity (FI) is intrinsically related not only to the lack of access to food but also to the continuous unavailability of food, a situation related to various everyday social deficiencies [1]. From this perspective, Brazil has approved a series of instruments and policies in the last two decades, aiming to guarantee the Human Right to Adequate Food (DHAA), among them the approval of the Organic Law of Food and Nutrition Security (LOSAN) in 2006 [2]. Despite this, 15 years after the DHAA was guaranteed among the social rights of the Federal Constitution of Brazil, in 2010, the right to be free from hunger and malnutrition and to have permanent access to adequate food is still not part of the absolute reality for a considerable proportion of the Brazilian people [3].

Thus, although Brazil left the world hunger map in 2014 due to social advances that improved the population’s quality of life, this progress was compromised in the following years [3,4]. In 2019, the dismantling of the National Council for Food and Nutrition Security (CONSEA) significantly weakened public policies aimed at food and nutrition security [4]. Shortly thereafter, the COVID-19 pandemic further aggravated this situation, both in Brazil and globally [3,4]. Without this government inspection and action program, the year 2020 brought, with the coronavirus pandemic, a significant increase in individuals with food insecurity due to the social restrictions of the period and the economic impact on the food supply, especially for the less affluent population [4,5].

In this regard, between 2021 and 2022 alone, more than 120 million Brazilians were exposed to food insecurity, with 33 million experiencing hunger [6]. Above all, one of the populations most affected by the pandemic was undergraduates. Undergraduates, in general, have an unhealthy diet, with a predominance of industrialized and highly processed foods, with little nutritional contribution and low intake of micronutrients, and this poor dietary quality is associated with the situation of food insecurity [7].

From this perspective, experiencing food insecurity can cause problems for undergraduates’ academic performance, causing this group to face psychological problems, such as stress and anxiety [8,9]. In this sense, it is essential to assess the situation of undergraduates during and after remote learning, given the usual vulnerability of this group to food insecurity and the worsening of this situation during the socioeconomic restrictions that characterized the period of this learning modality. In Brazil, after the implementation of the Quota Law in 2012—which allocates vacancies in undergraduate courses to students from public schools with a per capita family income of < 1.5, those on minimum wages, or those who self-declare as black, brown, or indigenous—there was a greater inclusion of students who were susceptible to situations of social vulnerability in public universities [10,11].

In addition, many students tend to leave their parents’ homes to go live alone near the university centers, which corroborates the lower purchasing power of Brazilian undergraduate students [12]. These students often depend on university restaurants to provide them with their main meals (which were suspended during remote learning) [13]. As the economic and political crisis worsened during the pandemic, this may have impacted this population’s regular access to nutritionally adequate food.

It is worth highlighting that permanent access to food is considered a fundamental condition for the maintenance of any individual’s life since food is an inalienable right [14]. Therefore, continually assessing constant and permanent access to food and balanced and adequate nutrition by undergraduates is essential to guarantee the quality of life of this group, as well as to preserve their social and human rights [14,15].

Data from a study conducted by José [7] at a Brazilian public university on the diet of undergraduates in the period after remote learning, more specifically in 2022, showed that undergraduates with higher socioeconomic status, non-quota students, and those who were in a food security situation consumed more natural or minimally processed foods. However, those in greater social vulnerability had a lower consumption of healthy foods. Despite this, the university restaurants (RUs) had a positive influence on the quality of food, as students who regularly ate their meals in these places tended to have a higher consumption of natural or minimally processed foods. In contrast, those who did not eat in these environments or resorted to commercial establishments presented less healthy eating habits.

The assessment carried out by [7] showed that situations related to social vulnerability influence current and habitual food consumption. Considering the vulnerability indicators used, approximately one-third of the students evaluated could be classified as socially vulnerable. Considering that food insecurity among undergraduates is a relevant social problem, with the potential to affect academic performance and the permanence of individuals in the institution until the end of the course, the importance of knowing the food and nutritional security situation in universities is reinforced, which corroborates the strengthening of access, assistance, and student retention policies to promote Food and Nutritional Security and the guarantee of the HRAA within the university food environment.

Although it is a consensus that undergraduates may be in situations of food insecurity [13,16,17,18,19,20], there is limited research on the changes in food security data that undergraduate students experience during and after remote learning. When universities changed their learning modality to remote learning, many students experienced changes in their lifestyles as well as in the socioeconomic situation of their households [16,21]. After remote learning, food insecurity situations among undergraduates may have improved due to the return to in-person learning and the potential improvement in the socioeconomic situation of these families. Because of this, it is necessary to understand food insecurity and the factors that were associated with it in undergraduates during and after remote learning.

Thus, the present study aimed to evaluate food insecurity and associated factors during and after remote learning among undergraduates at Brazilian public universities. The hypothesis of this comparative study is that food insecurity among undergraduates at public universities was lower in 2023 compared to 2020/2021 and associated with lower family incomes, reduced family income, worse health conditions, weight changes, worse diet qualities, and moderate-to-high perceived stress.

## 2. Materials and Methods

### 2.1. Study Design and Participants

This study is part of a multicenter project carried out at universities in four different states, covering four regions of Brazil: Acre (northern region), Rio Grande do Norte (northeast region), São Paulo (southeast region), and Paraná (south region). The project is entitled “Food Insecurity, Nutritional Status, and Lifestyle in the Academic Community during the COVID-19 Pandemic—BRAZUCA COVID” and was carried out at the Federal University of Acre—UFAC; Federal University of Rio Grande do Norte—UFRN; University of São Paulo—USP; and the Federal University of Paraná—UFPR.

This is a comparative study of two cross-sectional studies. Data collection took place in two stages, the first during remote learning carried out from August 2020 to February 2021, and the second stage after remote learning, from May 2023 to December 2023. Students regularly enrolled in undergraduate courses at public universities were eligible for the study.

An online questionnaire was created on the Google Forms platform and sent to the student’s institutional emails. Study participants were invited via institutional emails and/or social media. The questionnaire was a compilation of socioeconomic variables (including sex, family income, change in income during the pandemic and self-assessment of health status), self-reported weight changes, and the following scales validated in Portuguese: Brazilian Food Insecurity Scale (EBIA) [22,23], Diet Quality Scale [24], and Perceived Stress Scale [25,26], totaling 108 questions.

Volunteers who agreed to participate in the research answered the online questionnaire, without randomization in the sampling. Thus, the present research was carried out by non-probabilistic sampling.

Questionnaires with incomplete answers, defined as those in which the EBIA, ESQUADA and PSS scales that were not fully completed, were excluded from the sample. Inconsistent answers, characterized by data outside plausible standards, such as extremely high or low body weight values, incompatible with the typical body weight values, were also excluded.

**Figure 1:**
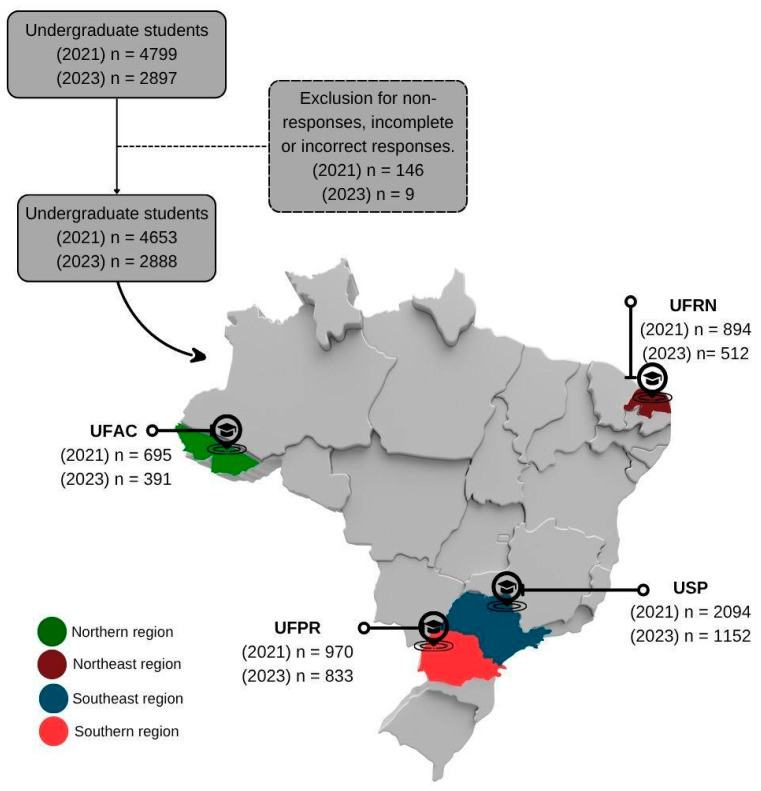
Flowchart of studied undergraduates (n = 7541) considering the study sites. UFAC—Federal University of Acre; UFRN—Federal University of Rio Grande do Norte; USP—University of São Paulo; UFPR—Federal University of Paraná.

A total of 7696 undergraduates from public universities responded to the questionnaire; 4799 responded in the first study, and 2897 responded in the second study. A total of 146 responses in the first study and 9 responses in the second, due to non-response, incomplete, or inconsistent responses, were excluded from this study, leaving a total of 7541 participants. In the first study, 695 undergraduate students were from UFAC, 894 from UFRN, 2094 from USP, and 970 from UFPR, totaling 4653 students. In the second study, 391 undergraduate students were from UFAC, 512 from UFRN, 1152 from USP, and 833 from UFPR, totaling 2888 students (Figure 1).

### 2.2. Food Insecurity

Food insecurity was assessed using the adapted and validated Brazilian Food Insecurity Scale (EBIA) (Cronbach’s alpha = 0.91) [22,23,27]. The scale is a direct measure of food insecurity, assessing the influence of money shortages on the availability and consumption of food by adults and children in their homes.

The application and analysis of the EBIA revealed that the scale captures common aspects of food insecurity in different sociocultural contexts. The aspects present in the scale include the following: (1) the psychological component, characterized by anxiety or uncertainty about the future availability of food for residents; (2) food quality, reflecting the restriction of cultural preferences and food variety in the household; (3) the reduction in the amount of food consumed by adults; (4) the reduction in children’s food intake; and (5) hunger, defined as the inability to eat for a whole day due to lack of financial resources to purchase food [28,29,30].

The scale is structured and calculated based on the sum of positive responses to 14 multiple-choice questions. The scale is constructed with dichotomous answers of “Yes” and “No”. The scores are organized within cutoff points that are equivalent to the theoretical constructs graded on food security [22].

Thus, the levels of food security were classified as food security (score 0); mild food insecurity (1 to 5 points for households with children under 18 and 1 to 3 points for households without children); moderate food insecurity (6 to 9 points for households with children and 4 to 5 points for those without children); and severe food insecurity (10 to 14 points for households with children and 6 to 8 points for those without children) [22].

### 2.3. Self-Referred Changes in Weight and Diet Quality Assessment

Self-referred changes in weight during the pandemic were registered in the online form. The diet quality scale (ESQUADA) [24] was applied to assess students’ diet quality. The ESQUADA presents 25 items on dietary practices (such as replacing meals with snacks and the habit of cooking) and the consumption of fresh, minimally processed, and ultra-processed food [24].

Item response theory (IRT) was applied to validate the ESQUADA, allowing the selection of items within a larger set of questions without compromising the accuracy of the score estimate, in accordance with the IRT invariance principle [31]. Based on this analysis, 15 items were chosen for this study, considering those with the greatest capacity to discriminate against diet quality, the greatest contribution to the definition of the ESQUADA levels, and without redundancy with other questions already present in the online questionnaire, either in terms of content or position on the scale.

The ESQUADA items include polytomous and dichotomous responses; however, all are ordinal, i.e., they have categories ordered according to alignment with the worst or best diet quality. The ESQUADA score was constructed by grouping into levels indicative of the cumulative trait of diet quality, obtaining the scores. These scores were analyzed and categorized into five levels of diet quality, using cutoff points from −2 to +2.5, as follows: “very poor” (scores ≤ −2); “bad” (scores > −2 and ≤−1), “good” (scores > −1 and ≤0.5); “very good” (scores > 0.5 and ≤2.5); and, “excellent” (scores > 2.5) [24].

### 2.4. Perceived Stress

Perceived stress was evaluated using the Perceived Stress Scale—PSS [25,32] a 10-item version validated for the Brazilian population (Cronbach’s alpha = 0.86) [26], and categorized as mild (scores ≤ 13), moderate (scores between 14 and 19), and high (scores ≥ 20) [32]. It is a self-reported measure that is designed to assess the degree to which situations in an individual’s life are assessed as stressful.

### 2.5. Statistical Analysis

The analyses were conducted using the Statistical Package for the Social Sciences SPSS^®^, version 26 (SPSS Inc. Chicago, IL, USA); the R software version 4.3.2 (R Core Team, Vienna, Austria) within the RStudio IDE (Posit, Boston, MA, USA); and Graph Pad Prism, version 8.0 (Graph Pad Software, San Diego, CA, USA).

Regarding the variables related to the characterization of the students, descriptive analyses were performed, testing normality with the Kolmogorov–Smirnov test to present the data as means (standard deviation) or medians and interquartile ranges (Q1–Q3). Quantitative variables were compared in the years 2020/2021 and 2023 using the Mann–Whitney U test to compare medians. Categorical variables were presented through frequency distribution, and associations were assessed using the Chi-square test. Given the large size of the sample studied, the significance level was set at *p* value < 0.001 in all tests used to minimize type 1 errors.

Correspondence analysis was conducted to explore the associations between food insecurity levels with variables that were significantly associated in the comparison of frequencies using the Chi-square test. This approach summarizes the information from categorical variables in a few dimensions, capturing the maximum variability contained in the variables included in the analysis, through the inertia calculated by the model. The objective of the analysis is to explain the greatest inertia or variation, with the fewest possible dimensions, which are calculated by the model. The assumptions of correspondence analysis are the homogeneity of variance between row and column variables, variables without zero entries, preferably with more than three categories, and without negative values. The results were represented in graphical maps, where each category of the variables was plotted as a point along the dimensions constructed by the analysis. The closer the points, the stronger the relationship between the categories [33].

Ordinal logistic regression models were constructed for variables that demonstrated an association in the bivariate analysis with food insecurity, giving further insights into variables associated with the different levels of food insecurity (mild, moderate, and severe). The effect of a single variable on food insecurity was first explored, and the unadjusted odds ratios (OR) and their respective 95% confidence intervals (95% CI) were presented. Then, for the multiple analysis, multicollinearity was tested for the independent variables, considering VIF values < 10, and tolerance > 0.1. The test of parallel lines was used, with a *p* value > 0.05 indicating the assumption held. The adjustment of the final model was also guaranteed by observing and an omnibus test and the test of model effects, with significant results (*p* < 0.05). The adjusted odds ratios (AOR) and their respective 95% CI were presented. Given the large sample size studied, the significance level was set at 1% to minimize type 1 errors.

## 3. Results

The characterization of the undergraduates is presented in Table 1. The median age was 22.0 years (20.0–26.0) in 2020/2021 and 23.0 years (20.0–30.0) in 2023, and the questionnaire was predominantly answered by women in both periods (66.1% in 2020/2021 and 64.9% in 2023). Most undergraduates were single (85.8% in 2020/2021 and 79.9% in 2023). Family income was mainly around 1 to 6 times the minimum wage. In 2020/2021 48.4% of undergraduates reported a reduction in family income in the last year during remote learning, while in 2023, 33.5% of students reported a reduction in income in the last year. In 2023 a higher percentage of students reported that family income remained unchanged in the last year (Table 1).

Considering weight change during the last year, 58.2% of undergraduates had an increase in weight during remote learning, and 51.8% reported weight gain in 2023. In both study periods, most students considered their current health status as fair or good (65.6% and 65.7%). On the other hand, during remote learning, 24.4% of the students reported poor or very poor health conditions, a higher percentage of students than in 2023. Considering perceived stress, 74.3% of the students presented elevated stress during the remote learning. In 2023, the percentage of students who self-assessed their perceived stress as high decreased to 70.8% (Table 1).

**Figure 2:**
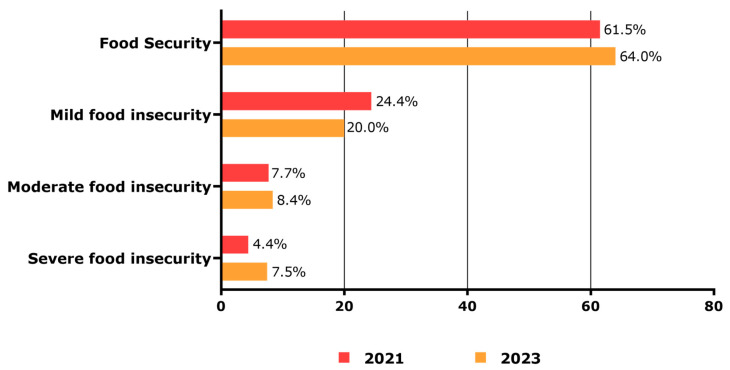
Food security situation of undergraduates in 2020/2021 and 2023.

When examining the food insecurity situation of undergraduates in the two periods, food security was present in 61.5% of the students in 2020/2021 and 64.0% in 2023. However, severe food insecurity was present in 4.4% of students in 2020/2021 and 7.5% in 2023, indicating a persistence of food insecurity even after remote learning (Figure 2).

**Figure 3:**
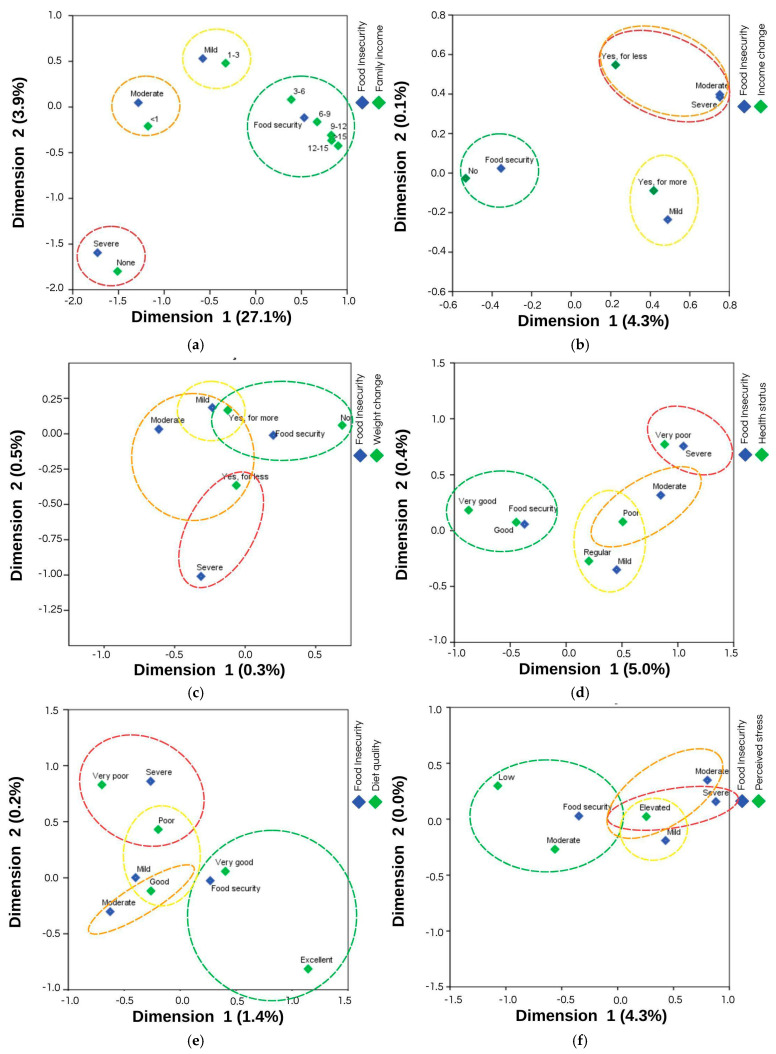
Correspondence maps between food insecurity, as assessed by the Brazilian Food Insecurity Scale and the factors that showed a significant association in the first phase of data collection, in 2020/2021: (**a**) correspondence map between food insecurity and family income in minimum wages, with a total model inertia of 31.2%; (**b**) correspondence map between food insecurity and income change, with a total model inertia of 4.4%; (**c**) correspondence map between food insecurity and weight change, with a total model inertia of 0.8%; (**d**) correspondence map between food insecurity and self-assessment health status, with a total model inertia of 6.3%; (**e**) correspondence map between food insecurity and diet quality, with a total model inertia of 1.6%; (**f**) correspondence map between food insecurity and perceived stress, with a total model inertia of 4.3%. Food insecurity classification: Mild Food Insecurity (Mild), Moderate Food Insecurity (Moderate) and Severe Food Insecurity (Severe).

Correspondence analyses indicated that in 2020/2021, students with lower family incomes (less than 3 times the minimum wage) were more closely associated with the levels of food insecurity, and family income explained 31.2% of food insecurity (Figure 3a). Students who had an increase in income during remote learning were closer to moderate and severe food insecurity, where the change in income accounted for 4.4% of food insecurity (Figure 3b). A reduction in body weight was strongly associated with severe food insecurity, with the change in weight explaining 0.8% of food insecurity (Figure 3c). Food insecurity levels were associated with poorer self-assessment health status, which accounted for 6.3% of food insecurity (Figure 3d). Better diet quality was associated with food security, while food insecurity levels were closer to worse diet qualities, explaining 1.6% of food insecurity (Figure 3e). Lastly, high perceived stress was close to all degrees of food insecurity, explaining 4.3% of the variance (Figure 3f).

**Figure 4:**
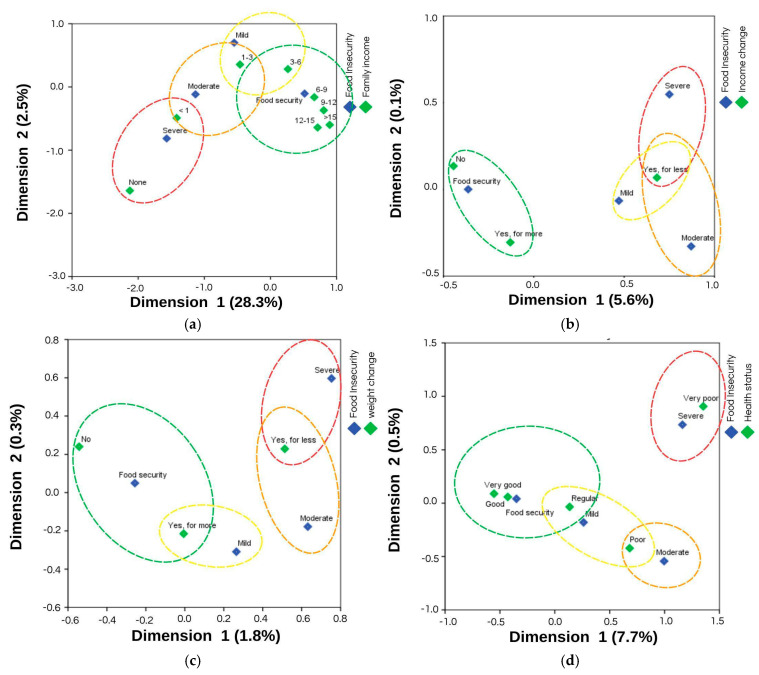

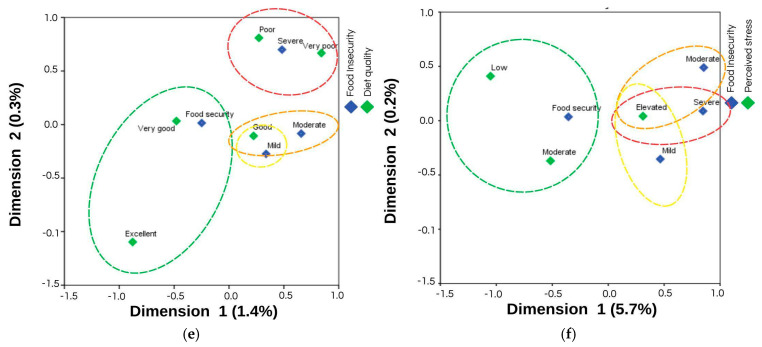
Correspondence maps between food insecurity, as assessed by the Brazilian Food Insecurity Scale and the factors that showed a significant association in the second phase of data collection, in 2023: (**a**) correspondence map between food insecurity and family income in minimum wages, with a total model inertia of 30.8%; (**b**) correspondence map between food insecurity and income change, with a total model inertia of 5.7%; (**c**) correspondence map between food insecurity and weight change, with a total model inertia of 2.0%; (**d**) correspondence map between food insecurity and self-assessment health status, with a total model inertia of 8.3%; (**e**) correspondence map between food insecurity and diet quality, with a total model inertia of 1.7%; (**f**) correspondence map between food insecurity and perceived stress, with a total model inertia of 5.9%. Food insecurity classification: Mild Food Insecurity (Mild), Moderate Food Insecurity (Moderate) and Severe Food Insecurity (Severe).

When analyzing the correspondence maps after the period of remote learning, in 2023, students from lower-income families (earning less than 4 times the minimum wage) were more strongly linked to food insecurity, with family income accounting for 30.8% of its variance (Figure 4a). Students who experienced a decrease in family income in 2023 showed a stronger correlation between all levels of food insecurity, with income change contributing to 5.7% of food insecurity (Figure 4b). A decrease in body weight was associated with moderate and severe food insecurity, with weight change explaining 2.0% of its variance (Figure 4c). Higher levels of food security were associated with better health self-assessment, which accounted for 8.3% of food insecurity (Figure 4d). Lower diet quality was closer to all levels of food insecurity, explaining 1.7% of its variance (Figure 4e). Finally, elevated perceived stress was strongly associated with all levels of food insecurity, contributing to 5.9% of its variance (Figure 4f).

Ordinal logistic regression analysis (Table 2) provided further insights into the observed associations. Students from lower-income families were significantly more likely to experience severe food insecurity, with an adjusted odds ratio (AOR) of 18.32 (95% CI–12.00–27.98). Maintaining the same income over the past year emerged as a protective factor against severe food insecurity, with an AOR of 0.73 (95% CI = 0.65–0.82). Similarly, students who did not experience weight changes during the past year were less likely to present severe food security, with an odds ratio (OR) of 0.75 (95% CI = 0.65–0.85).

Perceptions of better health status were also associated with lower odds of food insecurity: students who rated their health as “very good” had an AOR of 0.54 (95% CI = 0.40–0.71), while those who rated it as “good” had an AOR of 0.66 (95% CI = 0.53–0.84). Additionally, lower levels of perceived stress were associated with a lower likelihood of experiencing severe food insecurity, with students reporting low stress showing an AOR of 0.58 (95% CI = 0.47–0.74) and those with moderate stress an AOR of 0.73 (95% CI = 0.63–0.85). Lastly, whether students participated in the survey during (2020/2021) or after remote learning (2023) did not present a significant association with food insecurity.

## 4. Discussion

This study aimed to evaluate and compare food insecurity and its associated factors during and after remote learning among undergraduates at Brazilian public universities. Our data demonstrated a significant prevalence of food insecurity among university students, both during and after remote learning, with no significant difference between the two periods. This finding suggests that food insecurity is a problem that affects the undergraduates, and this problem persisted even after the pandemic and the end of emergency remote learning. In addition, it was significantly associated with household income, changes in income in the last year, weight changes during the last year, self-assessment health status, and perceived stress.

The COVID-19 pandemic has had a profound and lasting impact on food security, not only in Brazil but globally [6,34,35,36,37]. Studies have shown that food insecurity increased during the pandemic, especially among the most vulnerable populations. However, these effects have not been fully resolved even after the pandemic, as demonstrated by the data found in our study. The socioeconomic disruptions caused by the pandemic, such as job losses and food price inflation, which have reduced economic access to adequate food, may explain these results [38,39,40].

Food insecurity among Brazilian students is a significant issue, exacerbated by socioeconomic factors [16,19]. Our data indicate that both during and after remote learning, students from households earning less than three Brazilian minimum wages faced significantly higher odds of food insecurity. Socioeconomic factors play a significant role in the academic performance of college students. In a study conducted before the onset of remote learning, 94% of students who received financial aid faced food insecurity [19]. Another study conducted in São Paulo reported that 84.5% of university living in the University of São Paulo residential complex faced food insecurity, associated with insufficient income, eating less than three meals a day, and a lack of facilities for adequate meal preparation [13].

Not experiencing a change in household income in the past year was a protective factor against the levels of food insecurity. In the regression analysis, students who reported no change in family income were less likely to be food insecure. This reinforces the central role of income in the ability to access adequate food: poverty limits access to both nutritious foods and agricultural resources, making households more vulnerable to economic shocks and fluctuations in food prices [38]. Because of income limitations, many Brazilian families and undergraduates end up resorting to ultra-processed foods, which, although more affordable, are nutritionally poor and associated with worse health outcomes [17].

Food insecurity worsens poverty by harming health and productivity. This is because food insecurity leads to chronic hunger and malnutrition, which can affect the health status of individuals, thus reducing their productivity [21]. In this research, students who self-assessed their health status as poor or very poor were in a situation of moderate-to-severe food insecurity in both evaluated periods. In addition, self-assessment of health status as good or very good was significantly associated with food security, reducing the chances of being in a situation of food insecurity.

Another notable factor is that in both periods studied, the perception of high stress was associated with all levels of food insecurity. The relationship between mental health and food insecurity has a complex and bidirectional association [9,41,42,43,44] and its mechanisms are still not well understood. This relationship becomes even more complex when we consider the association between stress and food insecurity. Food insecurity is in itself a stressful life experience [45,46]. This may be explained by the fact that food insecurity is associated with an increase in mental health symptoms such as stress, depression and anxiety [43,44], whether due to the emotional distress caused by the lack of access to adequate and nutritious meals or due to its correlation with various other stressors linked to conditions of vulnerability and low socioeconomic status [45,46].

In a study of adults in the Czech Republic, adults with mental illness were 2.37 times more likely to be in a situation of food insecurity [47]. The literature also suggests that food insecurity can negatively impact mental health by depriving people of basic needs, such as constant uncertainty about the location and availability of their next meal [42].

Stress is a biopsychosocial phenomenon that involves both psychological and physiological responses [48]. When the hypothalamus detects stressful events, a physiological response occurs that leads to the release of cortisol [45,46,48]. This hormone, in turn, is linked to the increased consumption of hyperpalatable foods—rich in sugar, fat, and salt—as a form of emotional compensation [48].

Research shows that elevated cortisol levels resulting from chronic stress can affect glucose metabolism, especially among individuals experiencing food insecurity. This can exacerbate the effects of this condition, increasing the risk of developing chronic diseases such as diabetes and obesity [48]. This is an important aspect that warrants future investigation and the study of the mediating mechanisms that can explain how food insecurity exerts its effect on perceived stress.

It is also important to highlight that, after performing the ordinal logistic regression, the first or second period of data collection did not significantly contribute to food insecurity. This result indicates that food insecurity among university students at public universities in Brazil persisted even after the period of social isolation and the COVID-19 pandemic. Although the pandemic has impacted several socioeconomic aspects, such as the fact that several students experienced a reduction in income (almost 15% more during the period of remote teaching compared to the 2023, as demonstrated by the data in this study), food insecurity was already a pre-existing problem in this population [19] and may have worsened and persisted in the face of this public health emergency [49].

The eating habits observed among undergraduate students at public universities often deviate from the guidelines established for health promotion. This reality is strongly associated with the context of food insecurity experienced by many university students at these institutions [16,17,18]. According to data from the PENSSAN network, food insecurity in Brazil has intensified in recent years, especially during and after the COVID-19 pandemic. In 2021, 9% of Brazilians were in a situation of severe food insecurity [37], and in 2022 this percentage rose to 15.5%; 58.7% of the population still have some degree of food insecurity [6]. In 2023, the Food and Agriculture Organization of the United Nations (FAO) estimated that 70.3 million Brazilians lived in situations of moderate and severe food insecurity [36]. These data reflect a worsening of the national food situation, which corroborates the findings of the present study [36].

In addition, the constant increase in the price of food products can aggravate the effects of food insecurity. For example, at the end of 2022, the price of food rose by 37.5%, reducing the purchasing power of Brazilians for food [6,36,38,49]. Furthermore, the prices of essential food products, in the last 10 years, have increased proportionally more than the value of the minimum wage, reducing workers’ purchasing power and affecting their quality of life [50]. As ultra-processed foods are more affordable [50], undergraduates tend to favor purchasing these foods, which directly affect the quality of these individuals’ diets and their health [17].

Policies for access to higher education, such as the Quota Law, represent significant progress [10,11]. However, it is still difficult for students to remain in school due to various conditions, such as food insecurity. Undergraduates who come from families with lower incomes face difficulties in ensuring adequate nutrition, which can also compromise their academic performance. In this context, it is essential that, as well as guaranteeing access to higher education, public policies can strengthen student assistance programs, with an emphasis on initiatives that guarantee regular access to nutritionally adequate and quantitatively sufficient meals [51].

Some limitations of this study should be acknowledged. First, online data collection may have limited the participation of students who did not have good internet access. However, when the first data collection took place, Brazilian public universities already had support measures for students, such as grants for the acquisition of better-quality internet to ensure that classes could be attended during remote learning for the most vulnerable. During the second phase of data collection, students had already returned to in-person classes and could access the university’s internet to answer the questionnaire. Non-probabilistic sampling may have caused a selection bias, as the willingness to participate in the research may have been greater among students with higher levels of food insecurity. However, identifying and understanding these individuals was within the scope of the research. Using an online form leaves data collection more vulnerable to duplicate responses, but those duplicates from the same login were removed from this analysis, as demonstrated in Figure 1. Additionally, the reliance on self-reported data by students may have led to an underestimation or overestimation of the results. Although regional heterogeneity and differences in institutional support among Brazilian public universities may have influenced the results, it is essential to note that the study center was included as a covariable in the logistic regression models, which helped control for regional diversity in the analysis.

The strengths of this study are that we observed food insecurity during and after a period of remote learning that occurred in the face of a health emergency, also measuring diet quality, perceived stress, and socioeconomic factors concomitantly, which allowed for the observation of the demonstrated associations. Because this is a multicenter study, we obtained large samples from different regions of the country. Thus, the results can contribute to the planning of public policies for this group of individuals, who may be experiencing social, economic, mental, and health difficulties, due to the reality in which they find themselves, directly impacting their diet and education.

## 5. Conclusions

Our data showed a significant prevalence of food insecurity, both during and after remote learning, with no significant differences between the two periods. Food insecurity was associated with family income, income changes over the past year, weight changes, self-assessment health status, and perceived stress. These results suggest that food insecurity was already a pre-existing problem among undergraduates at Brazilian public universities and may have worsened and become chronic because of the COVID-19 pandemic and the transition from in-person to remote learning.

Our study highlights the need for targeted public policies for undergraduates directed at the most vulnerable: those with low family income, those who have experienced income changes, those who have weight changes, and those with a moderate and high self-perception of stress. Future research should focus on developing and evaluating programs that prioritize income generation and promote food security, nutritional education, and access to healthy foods within this population. Among the innovative strategies to combat food insecurity among undergraduate students, in addition to creating strategies to ensure access to proper food, would be the implementation of culinary workshops targeting university students. These workshops can be an effective tool for food and nutrition education, enabling students to prepare balanced, nutritious, and affordable meals with the resources available.

## Figures and Tables

**Table 1:** Sociodemographic and economic characteristics of undergraduates in 2020/2021 and 2023.

	2020/2021		2023		
	(n = 4653)		(n = 2888)		
Characteristics	Median (Q1–Q3)		Median (Q1–Q3)		U, *p*-value ^1^
Age	22.0 (20.0–26.0)		23.0 (20.0–30.0)		6,318,310, <0.001
Characteristics	N	%	N	%	χ^2^, *p*-value ^2^
Sex					
Male	1577	33.9	963	35.1	1.16, 0.282
Female	3076	66.1	1777	64.9	
Marital status					
Married	307	6.6	292	10.2	50.40, <0.001
Stable union	248	5.3	212	7.4	
Divorced	67	1.4	72	2.5	
Widower	6	0.1	1	0.0	
Single	4018	86.4	2288	79.9	
Family income in minimum wages ^3^					
None	127	2.7	25	0.9	101.68, <0.001
0–1	686	14.7	340	12.5	
1–3	1439	30.9	795	29.3	
3–6	1018	21.9	768	28.3	
6–9	515	11.1	367	13.5	
9–12	312	6.7	185	6.8	
12–15	207	4.4	58	2.1	
>15	311	6.7	178	6.6	
Family income change during the last year					
No	1907	41.0	1090	41.3	349.71, <0.001
Yes, for more	456	9.8	664	25.2	
Yes, for less	2249	48.3	884	33.5	
Change of address in the last year					
No	3424	73.6	2165	75.3	1.57, 0.210
Yes	1223	26.3	712	24.7	
Self-perceived weight change during the last year					
No	568	12.2	642	22.9	
Yes, for less	1285	27.6	711	25.3	122.19, <0.001
Yes, for more	2593	55.7	1453	51.8	
Self-assessment of health status					
Very good	458	9.8	368	12.7	36.02, <0.001
Good	1355	29.1	964	33.4	
Regular	1679	36.1	929	32.2	
Poor	877	18.8	474	16.4	
Very poor	263	5.7	152	5.3	
Diet quality assessment					
Very poor	63	1.3	24	0.8	1314.84, <0.001
Poor	406	8.6	171	5.9	
Good	2423	51.9	1757	41.4	
Very good	1779	37.1	926	32.1	
Excellent	52	1.1	4	0.1	
Perceived stress assessment					
Low	436	9.1	288	10.1	11.27, 0.004
Moderate	797	16.6	547	19.1	
Elevated	3566	74.3	2026	70.8	

^1^ Mann–Whitney U test; ^2^ Pearson’s Chi-square test; ^3^ In Brazil, the minimum wages were BRL 1100 in 2021, and BRL 1320 in 2023.

**Table 2:** Ordinal logistic regression for variables associated with food insecurity among undergraduates. BRAZUCA-COVID, 2020/2021 and 2023.

	Food Insecurity Classification			
Independent Variables	OR (95% CI)	*p* Value	AOR (95% CI)	*p* Value
Sex				
Male	0.84 (0.76–0.92)	<0.001	0.98 (0.88–1.10)	0.776
Female	-	-	-	-
Family income in minimum wages ^1^				
None	19.81 (13.42–29.24)	<0.001	18.32 (12.00–27.98)	<0.001
0–1	10.04 (7.62–13.24)	<0.001	9.15 (6.75–12.40)	<0.001
1–3	4.96 (3.81–4.45)	<0.001	4.55 (3.40–6.08)	<0.001
3–6	2.33 (1.78–3.05)	<0.001	2.17 (1.61–2.92)	<0.001
6–9	1.71 (1.28–2.31)	<0.001	1.69 (1.22–2.33)	0.001
9–12	1.09 (0.76–1.54)	0.633	1.13 (0.78–1.64)	0.515
12–15	0.75 (0.48–1.18)	0.211	0.79 (0.49–1.29)	0.351
>15	-	-	-	-
Family income change during the last year				
No	0.54 (0.49–0.60)	<0.001	0.73 (0.65–0.82)	<0.001
Yes, for more	0.83 (0.72–0.95)	0.008	0.94 (0.81–1.10)	0.474
Yes, for less	-	-	-	-
Self-perceived weight change during the last year				
No	0.75 (0.65–0.85)	<0.001	0.94 (0.80–1.10)	0.461
Yes, for less	0.97 (0.87–1.08)	0.607	1.06 (0.94–1.20)	0.312
Yes, for more	-	-	-	-
Self-assessment of health status				
Very good	0.30 (0.24–0.38)	<0.001	0.54 (0.40–0.71)	<0.001
Good	0.42 (0.35–0.52)	<0.001	0.66 (0.53–0.84)	<0.001
Regular	0.63 (0.52–0.77)	<0.001	0.74 (0.59–0.93)	0.008
Poor	0.80 (0.65–0.99)	0.043	0.82 (0.66–1.05)	0.120
Very poor	-	-	-	-
Diet quality assessment				
Very poor	2.38 (1.19–4.74)	0.014	1.17 (0.55–2.52)	0.682
Poor	1.29 (0.76–2.22)	0.340	0.76 (0.42–1.39)	0.371
Good	1.81 (1,07–3,07)	0.026	1.03 (0.57–1.85)	0.930
Very good	1.24 (0.73–2.12)	0.417	0.84 (0.46–1.50)	0.549
Excellent	-	-	-	-
Perceived stress assessment				
Low	0.39 (0.32–0.47)	<0.001	0.58 (0.47–0.74)	<0.001
Moderate	0.57 (0.49–0.65)	<0.001	0.73 (0.63–0.85)	<0.001
Elevated	-	-	-	-
Year of data collection				
2020/2021	1.02 (0.92–1.12)	0.694	0.92 (0.81–1.04)	0.195
2023	-	-	-	-

OR: crude odds ratio, from bivariate analysis; AOR: adjusted odds ratio, considering all variables in the model. Sex, age and study site were included as adjustment variables. ^1^ The minimum wages in Brazil are BRL 1100 (around BRL 212 higher than in 2021) and BRL 1320 (around BRL 249 higher than in 2023).

## Data Availability

The data presented in this study are available on request from the corresponding author.

## Abbreviations

The following abbreviations are used in this manuscript:

FI	Food insecurity
UFAC	Federal University of Acre
UFRN	Federal University of Rio Grande do Norte
USP	University of São Paulo
UFPR	Federal University of Paraná
OR	Odds ratio
AOR	Adjusted odds ratio
